# Supplementary material for: Dual Effect of Glucuronidation of a Pyrogallol-Type Phytophenol Antioxidant: A Comparison between Scutellarein and Scutellarin
Source: Molecules. 2018 Dec 6;23(12):3225. doi: 10.3390/molecules23123225 (PMC6321565; doi:10.3390/molecules23123225)
Supplement: Supplementary file 1 [file molecules-23-03225-s001.zip › molecules-399532-final-SM/Suppl. 2 Certificate analysis of Scutellarein.pdf]

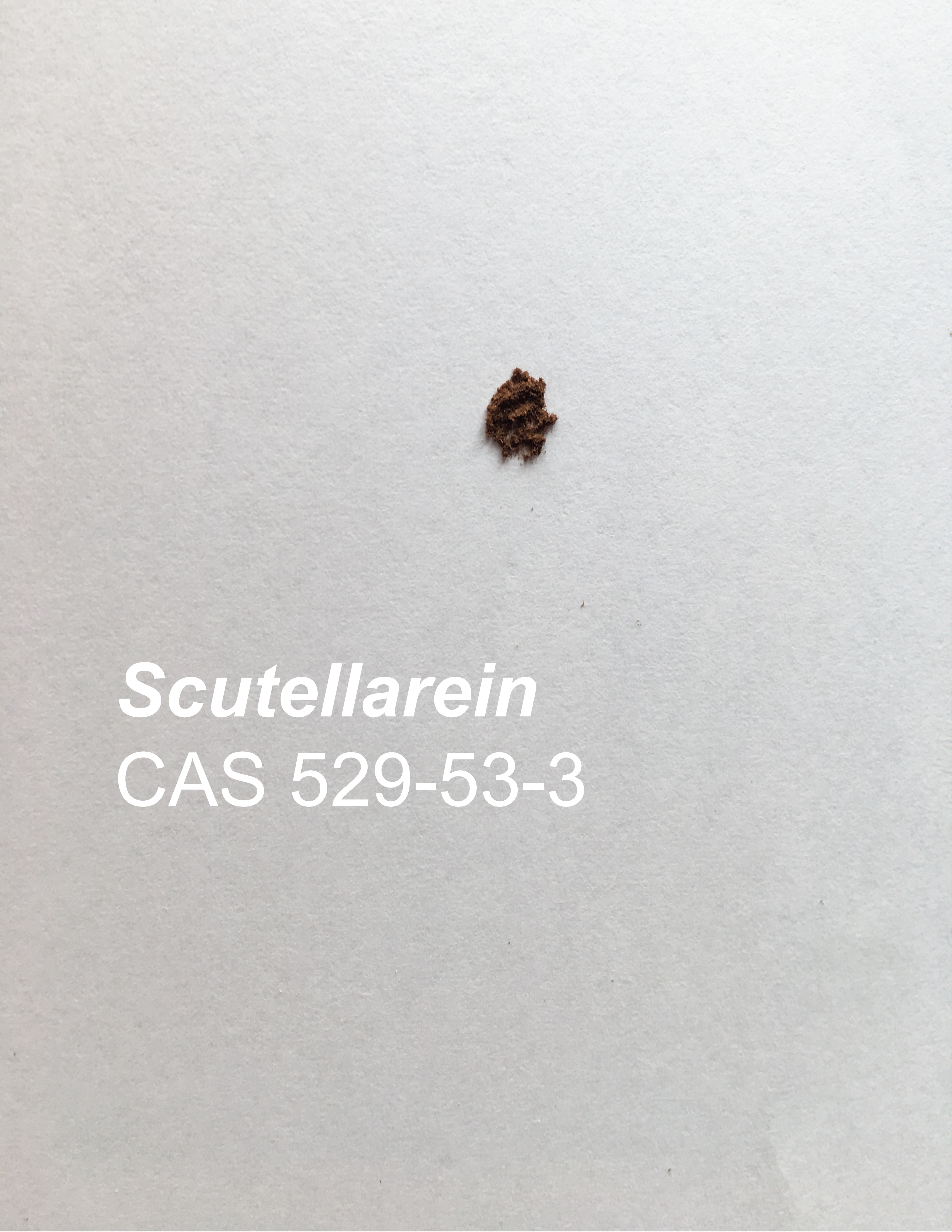

Scutellarein
CAS 529-53-3

西力生物
BBP BioBioPha
Natural Product Library for Drug Discovery

CERTIFICATE OF ANALYSIS

BBP No.: BBP04027

CAS No.: 529-53-3

Chemical Name: Scutellarein

Molecular Formula: $C_{15}H_{10}O_6$

Structure:

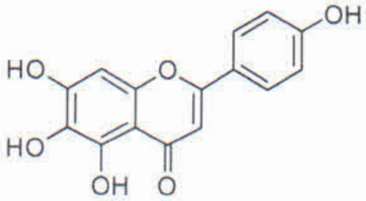

Purity: 98%

Appearance: Brown powder

Solvent: Dimethyl sulfoxide, methanol

Exact Weight: 5.1 mg

Storage: Store in a dark place under the temperature of 0–4 °C

Intended Use: For laboratory use only

Reference: H. J. Xia, Biol Pharm Bull, 2007, 30(7), 1308-1316

Warm Notice: When publishing, please cite as: ***chemical name*** was purchased from BioBioPha Co., Ltd. (Kunming, China)

Characterization Data Summary

Analytical Test	Results
Identification by ^{1}H-NMR	Consistent with the above structure
Purity tested by HPLC, ^{1}H-NMR	98%

Authorized Signature:

Date:

BioBioPha Co.,Ltd.
2017.11.29
PASS

Tel.: +86 871 6521 7109 Fax: +86 871 6521 5563 E-mail: sales@mail.biobiopha.com

西力生物
BBP BioBioPha

Natural Product Library for Drug Discovery

PRODUCT QUALITY REPORT

Product Number: BBP04027

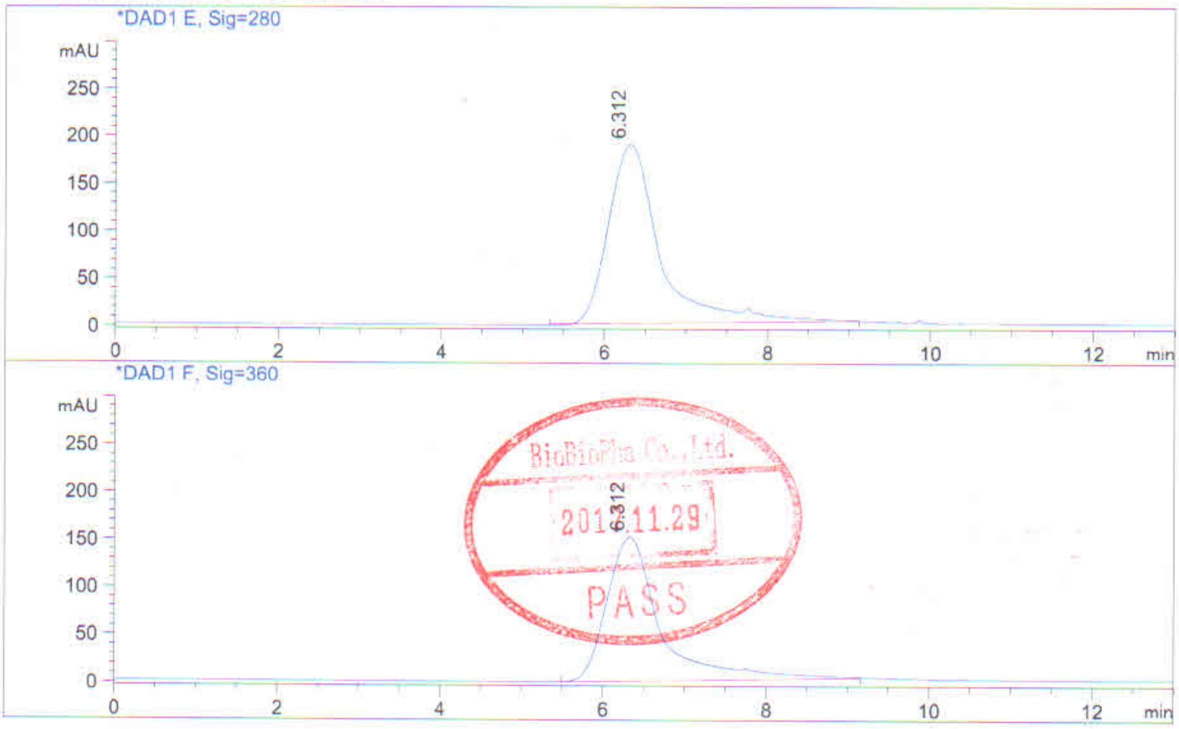

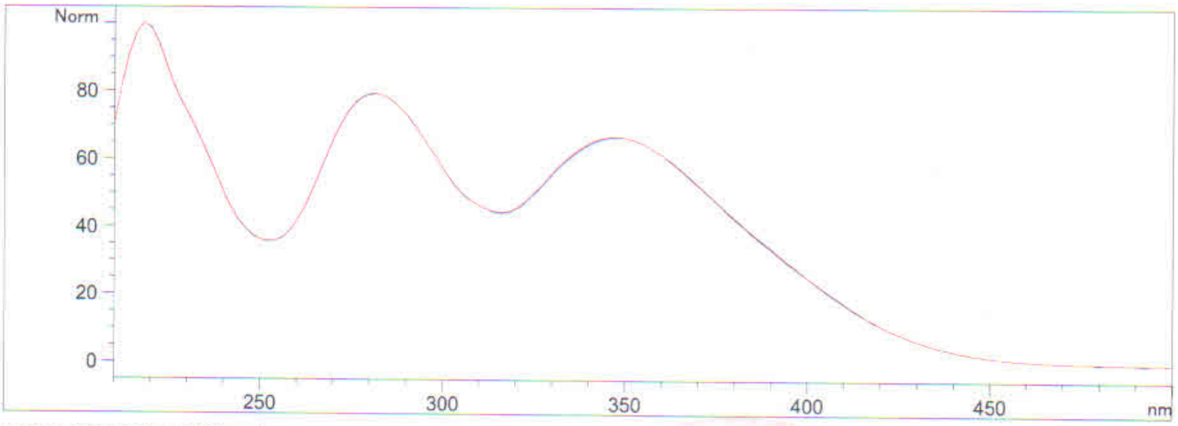

Agilent 1200 series HPLC system
Thermo Hypersil BDS C18 column (5 μm, 4.6 × 150 mm)
20%→100% MeOH in H_2O over 8.0 min followed by 100% MeOH to 13.0 min
1.0 ml/min, 25°C

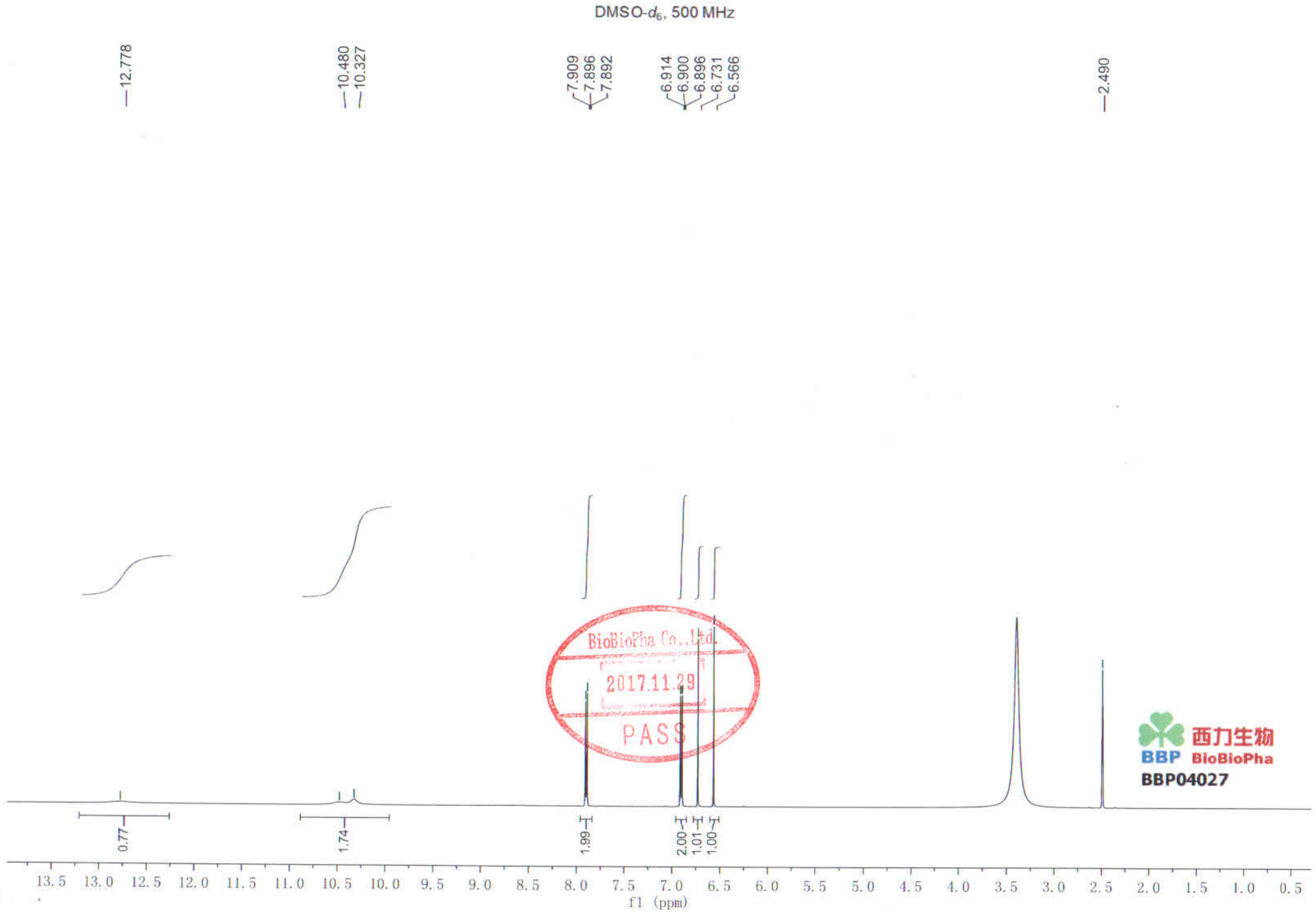
DMSO-d_6, 500 MHz
12.778
10.480
10.327
7.909
7.896
7.892
6.914
6.900
6.896
6.731
6.566
2.490
0.77
1.74
1.99
2.00
1.01
1.00
13.5 13.0 12.5 12.0 11.5 11.0 10.5 10.0 9.5 9.0 8.5 8.0 7.5 7.0 6.5 6.0 5.5 5.0 4.5 4.0 3.5 3.0 2.5 2.0 1.5 1.0 0.5
f1 (ppm)
BioBioPha Co., Ltd.
2017.11.29
PASS
西力生物
BBP BioBioPha
BBP04027
