# Supplementary material for: Dual Effect of Glucuronidation of a Pyrogallol-Type Phytophenol Antioxidant: A Comparison between Scutellarein and Scutellarin
Source: Molecules. 2018 Dec 6;23(12):3225. doi: 10.3390/molecules23123225 (PMC6321565; doi:10.3390/molecules23123225)
Supplement: Supplementary file 1 [file molecules-23-03225-s001.zip › molecules-399532-final-SM/Suppl. 3 Certificate analysis of Scutellarin.pdf]

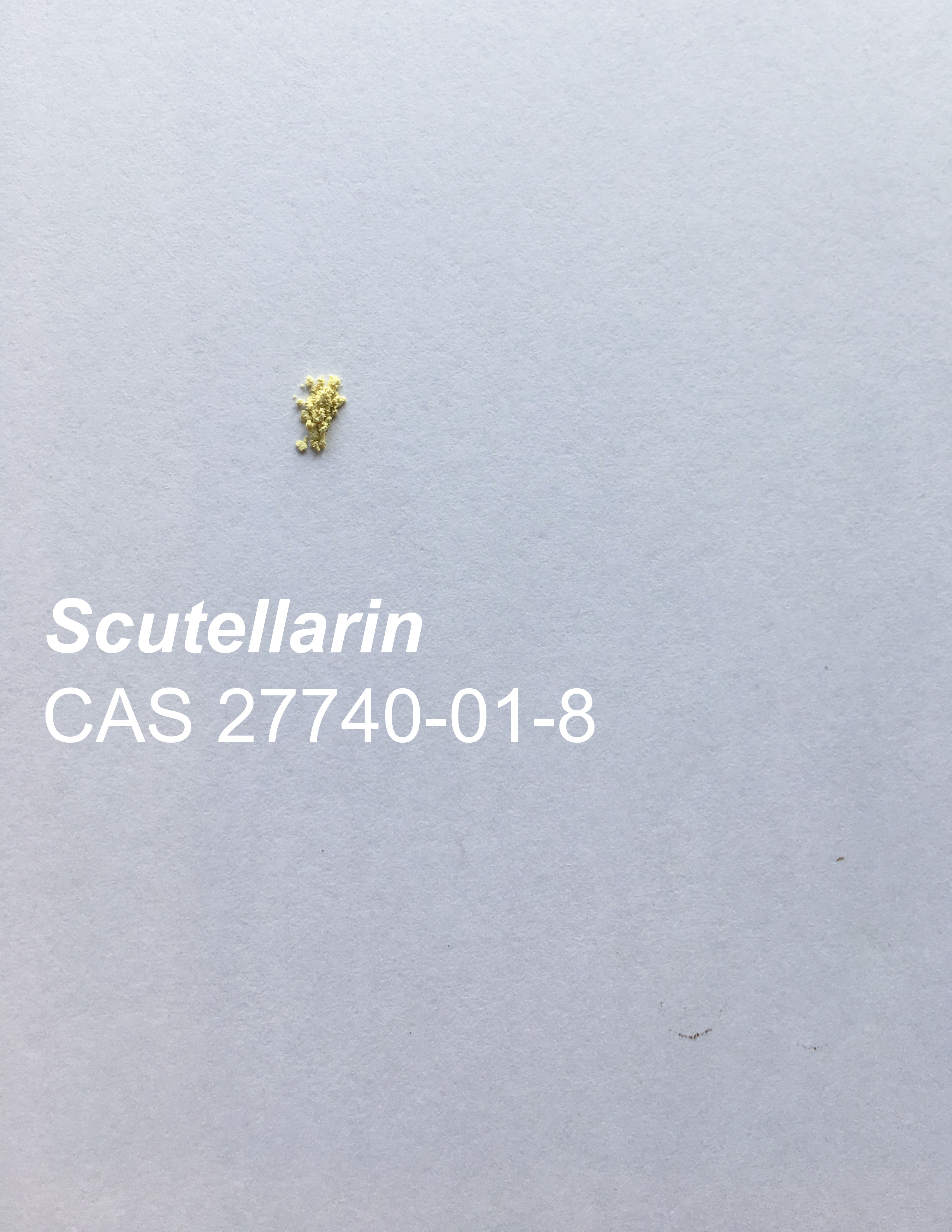
Scutellarin
CAS 27740-01-8

西力生物
BBP BioBioPha
Natural Product Library for Drug Discovery

CERTIFICATE OF ANALYSIS

BBP No.: BBP03864

CAS No.: 27740-01-8

Chemical Name: Scutellarin

Molecular Formula: $C_{21}H_{18}O_{12}$

Structure:

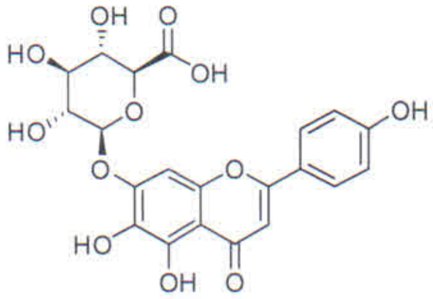

Purity: 98%

Appearance: Yellow powder

Solvent: Dimethyl sulfoxide, methanol

Exact Weight: 5.1 mg

Storage: Store in a dark place under the temperature of 0−4 °C

Intended Use: For laboratory use only

Reference: J. Lu, Eur J Med Chem, 2010, 45(5), 1731-1738

Warm Notice: When publishing, please cite as: ***chemical name*** was purchased from BioBioPha Co., Ltd. (Kunming, China)

Characterization Data Summary

Analytical Test	Results
Identification by ^{1}H-NMR	Consistent with the above structure
Purity tested by HPLC, ^{1}H-NMR	98%

Authorized Signature: BioBioPha Co., Ltd. 2017.11.29 PASS

Date:

Tel.: +86 871 6521 7109 Fax: +86 871 6521 5563 E-mail: sales@mail.biobiopha.com

西力生物
BBP BioBioPha

Natural Product Library for Drug Discovery

PRODUCT QUALITY REPORT

Product Number: BBP03864

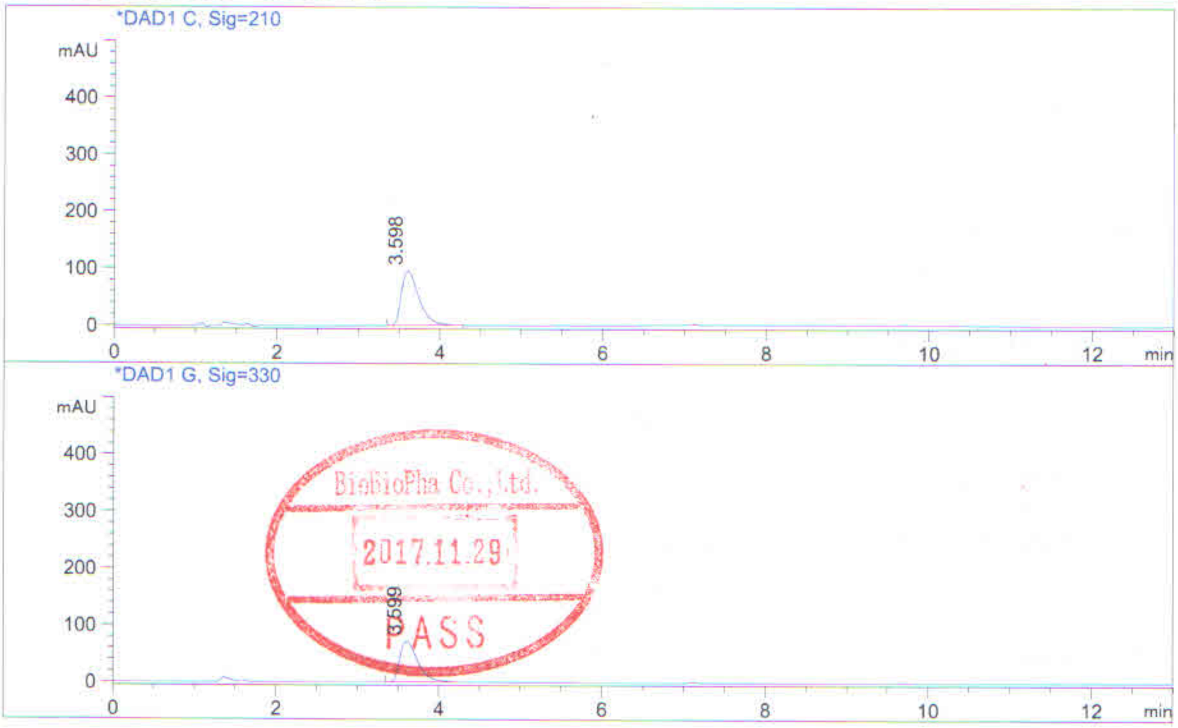

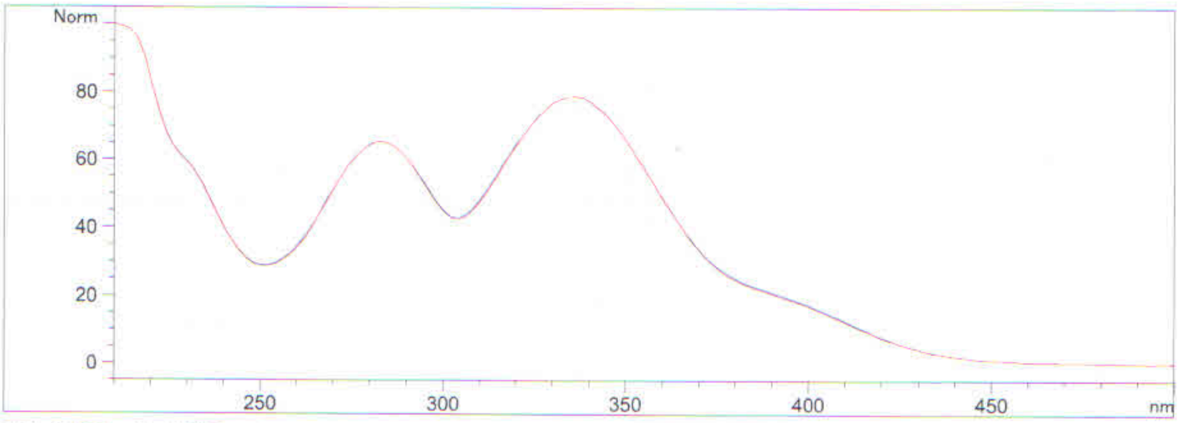

Agilent 1200 series HPLC system
Extend-C18 column (5 μm, 4.6 × 150 mm)
20%→100% MeOH in H_2O over 8.0 min followed by 100% MeOH to 13.0 min
1.0 ml/min, 20°C

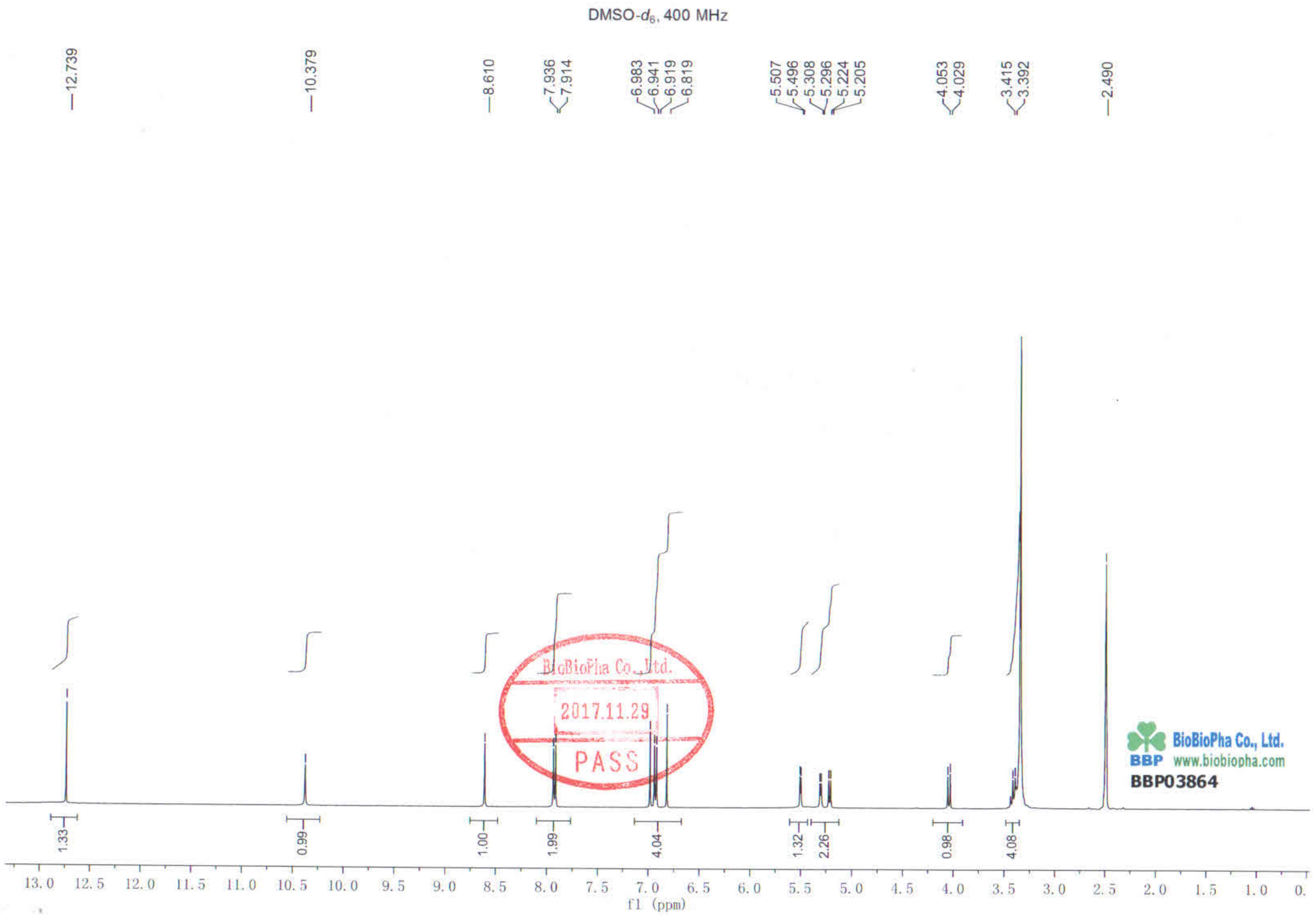
DMSO-d_6, 400 MHz
12.739
10.379
8.610
7.936
7.914
6.983
6.941
6.919
6.819
5.507
5.496
5.308
5.296
5.224
5.205
4.053
4.029
3.415
3.392
2.490
BioBioPha Co., Ltd.
2017.11.29
PASS
BioBioPha Co., Ltd.
BBP www.biobiopha.com
BBP03864
1.33
0.99
1.00
1.99
4.04
1.32
2.26
0.98
4.08
13.0
12.5
12.0
11.5
11.0
10.5
10.0
9.5
9.0
8.5
8.0
7.5
7.0
6.5
6.0
5.5
5.0
4.5
4.0
3.5
3.0
2.5
2.0
1.5
1.0
0.
f1 (ppm)
