# Supplementary material for: Dual Effect of Glucuronidation of a Pyrogallol-Type Phytophenol Antioxidant: A Comparison between Scutellarein and Scutellarin
Source: Molecules. 2018 Dec 6;23(12):3225. doi: 10.3390/molecules23123225 (PMC6321565; doi:10.3390/molecules23123225)
Supplement: Supplementary file 1 [file molecules-23-03225-s001.zip › molecules-399532-final-SM/Suppl.1.pdf]

Suppl. 1 Dose response curves Figure S1-S5

Dual Effect of Glucuronidation towards Antioxidant Pyrogallol-pattern Phytophenol: A Comparison between Scutellarein and Scutellarin

Qianru Liu [1,2,†], Xican Li[1,2,†,*], Xiaojian Ouyang [1,2], and Dongfeng Chen [3,4,*]

[1] School of Chinese Herbal Medicine; Guangzhou University of Chinese Medicine, Guangzhou 510006, China. E-mails: liuqianru2333@163.com (L.Q);

[2] Innovative Research & Development Laboratory of TCM; Guangzhou University of Chinese Medicine, Guangzhou 510006, China.

[3] School of Basic Medical Science, Guangzhou University of Chinese Medicine, Guangzhou, China, 510006

[4] The Research Center of Basic Integrative Medicine, Guangzhou University of Chinese Medicine, Guangzhou, China, 510006. E-mail: chen888@gzucm.edu.cn (D.C.)

* Correspondence: lixc@gzucm.edu.cn (X.L.); lixican@126.com (X.L.); chen888@gzucm.edu.cn (D.C.) Tel: +86-20-39358076; Fax: +86-20-38892690

† These authors contributed equally to this work.

Note: This Supporting information provides the original data of **Table 1** in the main text. All data underline are mentioned in **Table 1** in the main text.

1. *DPPH•-scavenging assay*

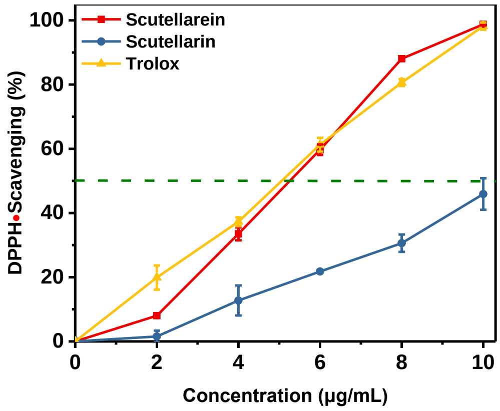

Figure S1: The dose response curves of scutellarein and scutellarin in DPPH•-scavenging assay. Each value is expressed as mean ± SD (n = 3).

Tab. S1 The comparison of IC_{50} values of scutellarein and scutellarin and positive control in DPPH•-scavenging assay.

	Mean±SD μg/mL	Mean±SD μM
scutellarein	5.4 ± 0.0	18.7 ± 0.1 [a]
scutellarin	11.2 ± 0.8	24.2 ± 1.7 [b]
Trolox	5.0 ± 0.1	20.2 ± 0.5 [a]

IC_{50} value was defined as the concentration of 50% superoxide anion radical inhibition and calculated by linear regression which was analyzed by Origin 6.0 professional software. Means values with different superscripts in the same column are significantly different ($p<0.05$).

2. *ABTS^{+}•-scavenging assay*

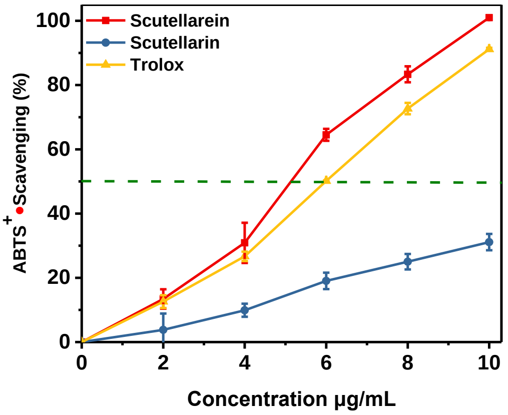

Figure S2: The dose response curves of scutellarein and scutellarin in ABTS^{+}•-scavenging assay. Each value is expressed as mean ± SD (n = 3).

Tab. S2 The comparison of IC_{50} values of scutellarein and scutellarin and positive control in ABTS+•-scavenging assay.

	Mean±SD μg/mL	Mean±SD μM
scutellarein	5.2 ± 0.3	18.3 ± 1.2 [a]
scutellarin	15.4 ± 1.3	33.3 ± 2.9 [c]
Trolox	5.9 ± 0.1	23.7 ±0.4 [b]

IC_{50} value was defined as the concentration of 50% superoxide anion radical inhibition and calculated by linear regression which was analyzed by Origin 6.0 professional software. Means values with different superscripts in the same column are significantly different ($p<0.05$).

3. *PTIO•-scavenging assay*

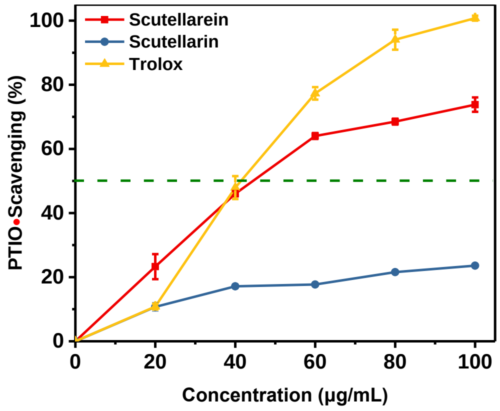

Figure S3: The dose response curves of scutellarein and scutellarin in PTIO•-scavenging assay. Each value is expressed as mean ± SD (n = 3).

Tab. S3 The comparison of IC_{50} values of scutellarein and scutellarin and positive control in PTIO•-scavenging assay.

	Mean±SD μg/mL	Mean±SD μM
scutellarein	50.8 ± 2.2	177.5 ± 7.8 [a]
scutellarin	266.9 ±34.8	577.2 ± 75.4 [b]
Trolox	46.5 ±2.3	185.7 ± 9.0 [a]

IC_{50} value was defined as the concentration of 50% superoxide anion radical inhibition and calculated by linear regression which was analyzed by Origin 6.0 professional software. Means values with different superscripts in the same column are significantly different ($p<0.05$).

4. *Cu^{2+}-reducing power assay*

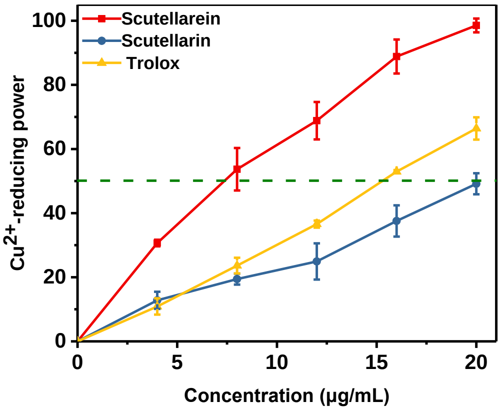

Figure S4: The dose response curves of scutellarein and scutellarin in Cu^{2+}-reducing power assay. Each value is expressed as mean ± SD (n = 3).

Tab. S4 The comparison of IC_{50} values of scutellarein and scutellarin and positive control in Cu^{2+}-reducing power assay.

	Mean±SD µg/mL	Mean±SD µM
scutellarein	9.6 ± 0.4	33.5 ± 1.4 [a]
scutellarin	20.1 ± 0.7	43.4 ±1.5 [b]
Trolox	15.4 ± 0.5	61.5 ± 2.0[c]

IC_{50} value was defined as the concentration of 50% superoxide anion radical inhibition and calculated by linear regression which was analyzed by Origin 6.0 professional software. Means values with different superscripts in the same column are significantly different ($p<0.05$).

5. *Superoxide anion ($\bullet O_2^-$) scavenging assay*

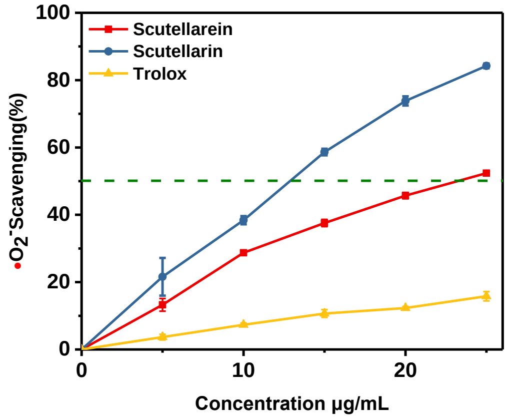

Figure S5: The dose response curves of scutellarein and scutellarin in Superoxide anion ($\bullet O_2^-$) scavenging assay. Each value is expressed as mean ± SD (n = 3).

Tab. S5 The comparison of IC_{50} values of scutellarein and scutellarin and positive control in Superoxide anion ($\bullet O_2^-$) scavenging assay.

	Mean±SD<br>μg/mL	Mean±SD<br>μM
scutellarein	22.6 ± 0.2	79.0 ± 0.5 [b]
scutellarin	13.3 ± 0.6	28.8 ±1.4 [a]
Trolox	83.4 ±11.6	291.5 ±40.6 [c]

IC_{50} value was defined as the concentration of 50% superoxide anion radical inhibition and calculated by linear regression which was analyzed by Origin 6.0 professional software. Means values with different superscripts in the same column are significantly different ($p<0.05$).
